# Markers of fungal translocation are elevated during post-acute sequelae of SARS-CoV-2 and induce NF-**κ**B signaling

**DOI:** 10.1172/jci.insight.160989

**Published:** 2022-08-08

**Authors:** Leila B. Giron, Michael J. Peluso, Jianyi Ding, Grace Kenny, Netanel F. Zilberstein, Jane Koshy, Kai Ying Hong, Heather Rasmussen, Gregory E. Miller, Faraz Bishehsari, Robert A. Balk, James N. Moy, Rebecca Hoh, Scott Lu, Aaron R. Goldman, Hsin-Yao Tang, Brandon C. Yee, Ahmed Chenna, John W. Winslow, Christos J. Petropoulos, J. Daniel Kelly, Haimanot Wasse, Jeffrey N. Martin, Qin Liu, Ali Keshavarzian, Alan Landay, Steven G. Deeks, Timothy J. Henrich, Mohamed Abdel-Mohsen

**Affiliations:** 1 The Wistar Institute, Philadelphia, Pennsylvania, USA.; 2 UCSF, San Francisco, California, USA.; 3 Centre for Experimental Pathogen Host Research, University College Dublin, Dublin, Ireland.; 4 Department of Internal Medicine, Rush University, Chicago, Illinois, USA.; 5 University of Nebraska–Lincoln, Lincoln, Nebraska, USA.; 6 Northwestern University, Evanston, Illinois, USA.; 7 Rush Center for Integrated Microbiome and Chronobiology Research, Rush University, Chicago, Illinois, USA.; 8 Monogram Biosciences, Inc., Labcorp, South San Francisco, California, USA.

**Keywords:** COVID-19, Virology, Tight junctions

## Abstract

Long COVID, a type of post-acute sequelae of SARS-CoV-2 (PASC), has been associated with sustained elevated levels of immune activation and inflammation. However, the mechanisms that drive this inflammation remain unknown. Inflammation during acute coronavirus disease 2019 could be exacerbated by microbial translocation (from the gut and/or lung) to blood. Whether microbial translocation contributes to inflammation during PASC is unknown. We did not observe a significant elevation in plasma markers of bacterial translocation during PASC. However, we observed higher levels of fungal translocation — measured as β-glucan, a fungal cell wall polysaccharide — in the plasma of individuals experiencing PASC compared with those without PASC or SARS-CoV-2–negative controls. The higher β-glucan correlated with higher inflammation and elevated levels of host metabolites involved in activating *N*-methyl-d-aspartate receptors (such as metabolites within the tryptophan catabolism pathway) with established neurotoxic properties. Mechanistically, β-glucan can directly induce inflammation by binding to myeloid cells (via Dectin-1) and activating Syk/NF-κB signaling. Using a Dectin-1/NF-κB reporter model, we found that plasma from individuals experiencing PASC induced higher NF-κB signaling compared with plasma from negative controls. This higher NF-κB signaling was abrogated by piceatannol (Syk inhibitor). These data suggest a potential targetable mechanism linking fungal translocation and inflammation during PASC.

## Graphical abstract

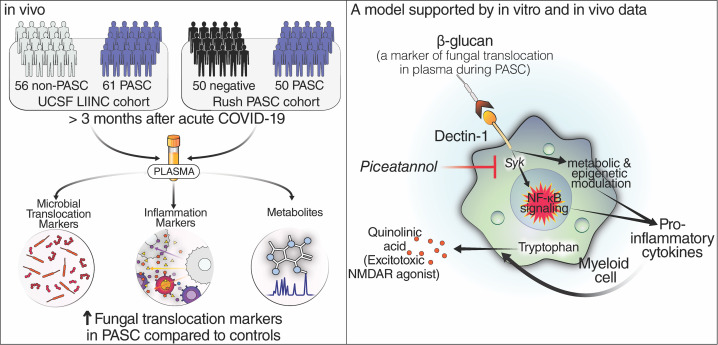

## Introduction

SARS-CoV-2 infection causes acute respiratory and systemic disease (coronavirus disease 2019, COVID-19) (1, 2). A subset of individuals also experience persistent, recurrent, or new COVID-19–attributed symptoms in the months following acute infection — a condition commonly referred to as long COVID (3–19). Long COVID, a type of post-acute sequelae of SARS-CoV-2 infection (PASC), can affect an individual’s overall health and quality of life (3–18, 20). Recently, PASC has been associated with sustained elevated levels of immune activation and inflammation (21–24). However, the pathophysiological mechanisms that drive this inflammation remain unknown. Among the hypothesized drivers are preexisting medical comorbidities, such as diabetes or obesity, the degree of SARS-CoV-2 viremia during acute infection, latent Epstein-Barr virus reactivation, and the production of autoantibodies (25–27). We have been investigating a known driver of systemic inflammation and severity during other respiratory diseases, microbial translocation resulting from disruption in the gut-lung axis.

Disruption of the gut-lung axis is a known marker of severity during other respiratory diseases (28–31) and may play a role in potentiating worse clinical outcomes. SARS-CoV-2 infection can affect the gastrointestinal tract (GI) tract and cause GI symptoms (32, 33) through indirect and/or direct mechanisms. Indirectly, lung infection or injury can provoke systemic inflammation (including cytokine storm), which in turn can disrupt gut barrier integrity (mainly by IFN-γ and TNF-α, which are known to disrupt tight junction permeability; refs. 34–36), enabling gut microbes and their products to translocate across the gut epithelium. This translocation (which can also happen across the lung epithelium) can exacerbate the initial systemic inflammation, resulting in a positive feedback loop (28–31, 37–39). Directly, SARS-CoV-2 can infect gut cells (40); other viral infections of the gut can cause a breakdown of the epithelial barrier (41–43).

The human microbiome is composed of bacteria, fungi, protozoa, and viruses (44–46). Microbial translocation of bacteria and bacterial products (such as LPS) and the subsequent immune activation and inflammation are well documented (47–49). However, there is emerging evidence that fungal translocation also plays an important role in driving immune activation and inflammation in conditions involving epithelial barrier permeability (such as HIV infection) (50, 51). Translocated fungal products induce immune activation and inflammation by binding to their receptors on the surface of immune cells, including macrophages, monocytes, and dendritic cells, to induce proinflammatory signaling pathways (reviewed in ref. 52).

Acute COVID-19 has been associated with an increase in the plasma levels of zonulin, an established physiological driver of tight junction permeability (53, 54). This increased permeability enables the translocation of both bacterial and fungal products to the blood. Such microbial translocation correlates with increased systemic inflammation, disrupted gut-associated metabolites, and higher mortality during acute COVID-19 (55). These observations are supported with a series of recent studies, using stool samples, showing that COVID-19 severity is associated with a state of gut microbial dysbiosis and translocation (including fungal translocation) (56–63). Although these data (55–62) do not imply that gut microbial translocation is the primary trigger of inflammation during COVID-19, as the clinical syndrome of COVID-19 likely embodies multiple pathophysiological pathways, they are consistent with the literature indicating that microbial translocation fuels inflammation and disease severity during respiratory diseases (28–31) and thus support a model in which microbial translocation fuels inflammation following SARS-CoV-2 infection. However, whether the translocation of microbes — bacteria or fungus — is related to inflammation during PASC is unknown and is the subject of this study.

## Results

### Participant characteristics.

We used samples from 2 cohorts (Table 1): 1) Cross-sectional plasma samples from 117 volunteers with a history of COVID-19 (a subset of the UCSF LIINC cohort) 90–160 days after their first positive SARS-CoV-2 result. These participants were divided into 2 groups: 56 individuals with no ongoing COVID-19–attributed symptoms at the time of sample collection (non-PASC) and 61 with at least 2 symptoms present at the time of sample collection (PASC; Table 1). 2) Cross-sectional plasma samples from 50 COVID-19 individuals experiencing PASC 3–4 months after their convalescence from acute COVID-19 (a subset of the Rush PASC cohort) and cross-sectional plasma samples from 50 SARS-CoV-2–negative controls (matched for age and sex to the Rush PASC samples; Table 1).

**Figure 1:**
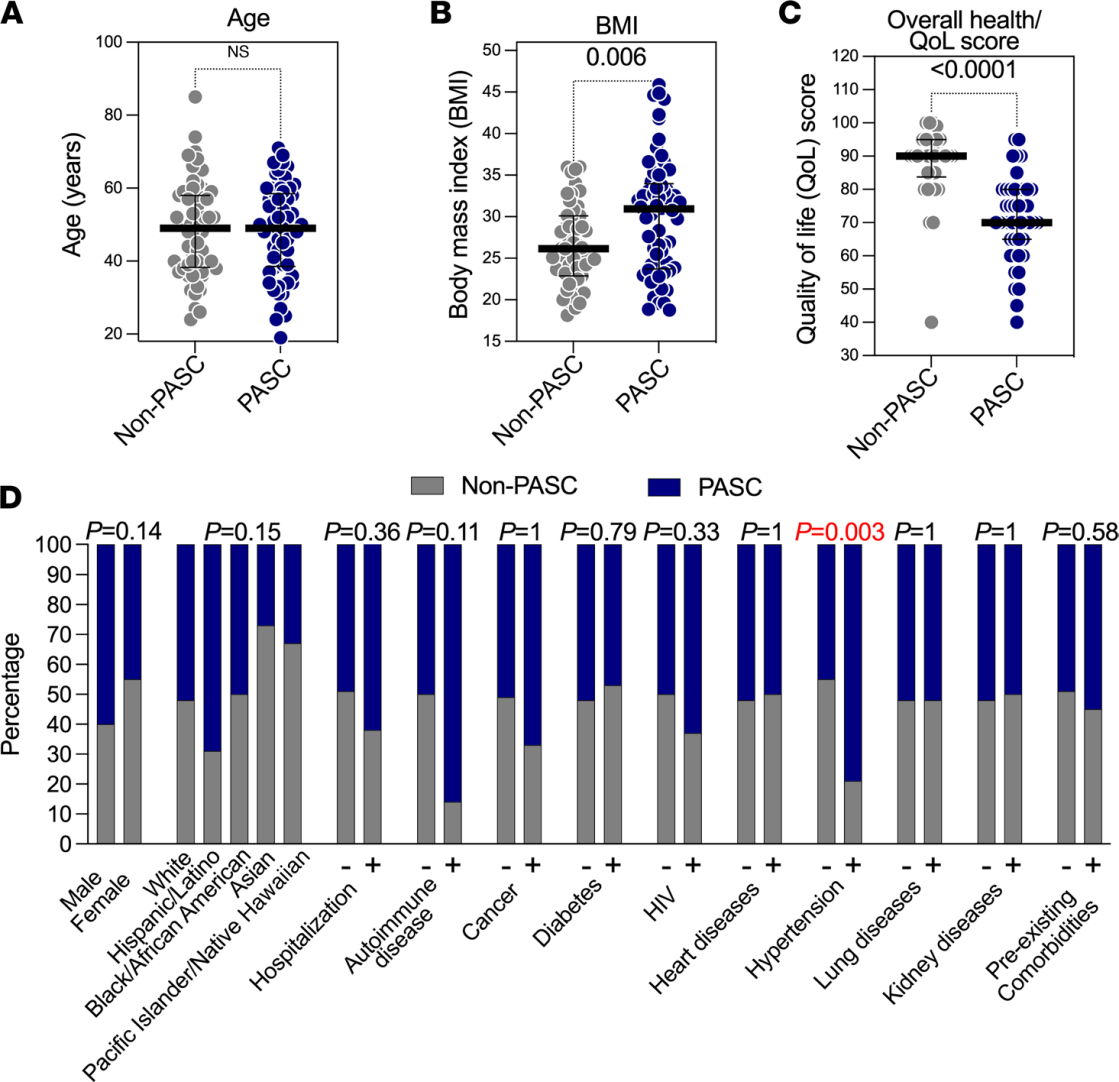
BMI and hypertension differentiate PASC from non-PASC in a subset of the UCSF LIINC cohort. Demographic and clinical characteristics of 117 individuals from the UCSF LIINC cohort. Out of these 117 individuals, 61 individuals were experiencing 2 or more COVID-19–attributed symptoms 4 months following SARS-CoV-2 infection (PASC), whereas 56 individuals were not experiencing any ongoing symptoms (non-PASC). (**A**–**C**) Mann-Whitney *U* comparisons of (**A**) age, (**B**) BMI, and (**C**) overall health/quality of life (QoL) score between PASC and non-PASC within the 117 individuals from the UCSF LIINC cohort. Median and IQR are displayed. (**D**) Fisher’s exact test comparisons of the indicated demographic and clinical characteristics between PASC and non-PASC within the 117 individuals from the UCSF LIINC cohort. + = yes; — = no.

We examined whether age (Figure 1A), BMI (Figure 1B), self-rated overall health/quality of life (QoL) score on a visual analog scale (0–100; Figure 1C), sex, ethnicity, hospitalization during acute COVID-19, or preexisting comorbidities (Figure 1D) differentiated PASC from non-PASC groups within the 117 samples from the UCSF LIINC cohort. We found that a higher BMI (*P* = 0.006; Figure 1B) and a higher rate of preexisting hypertension (*P* = 0.003; Figure 1D) were associated with the PASC phenotype. The overall health/QoL score was lower in volunteers experiencing PASC than those in the non-PASC group (*P* < 0.0001; Figure 1C). Based on these observations, we adjusted our subsequent analyses on the potential role of microbial translocation in PASC for BMI and hypertension as potential confounders of the PASC phenotype in this subset from the UCSF LIINC cohort. We also used the overall health/QoL score in our subsequent analyses examining the potential impact of microbial translocation on individuals’ well-being during PASC.

### PASC is associated with elevated levels of fungal translocation independent of BMI and hypertension.

**Figure 2:**
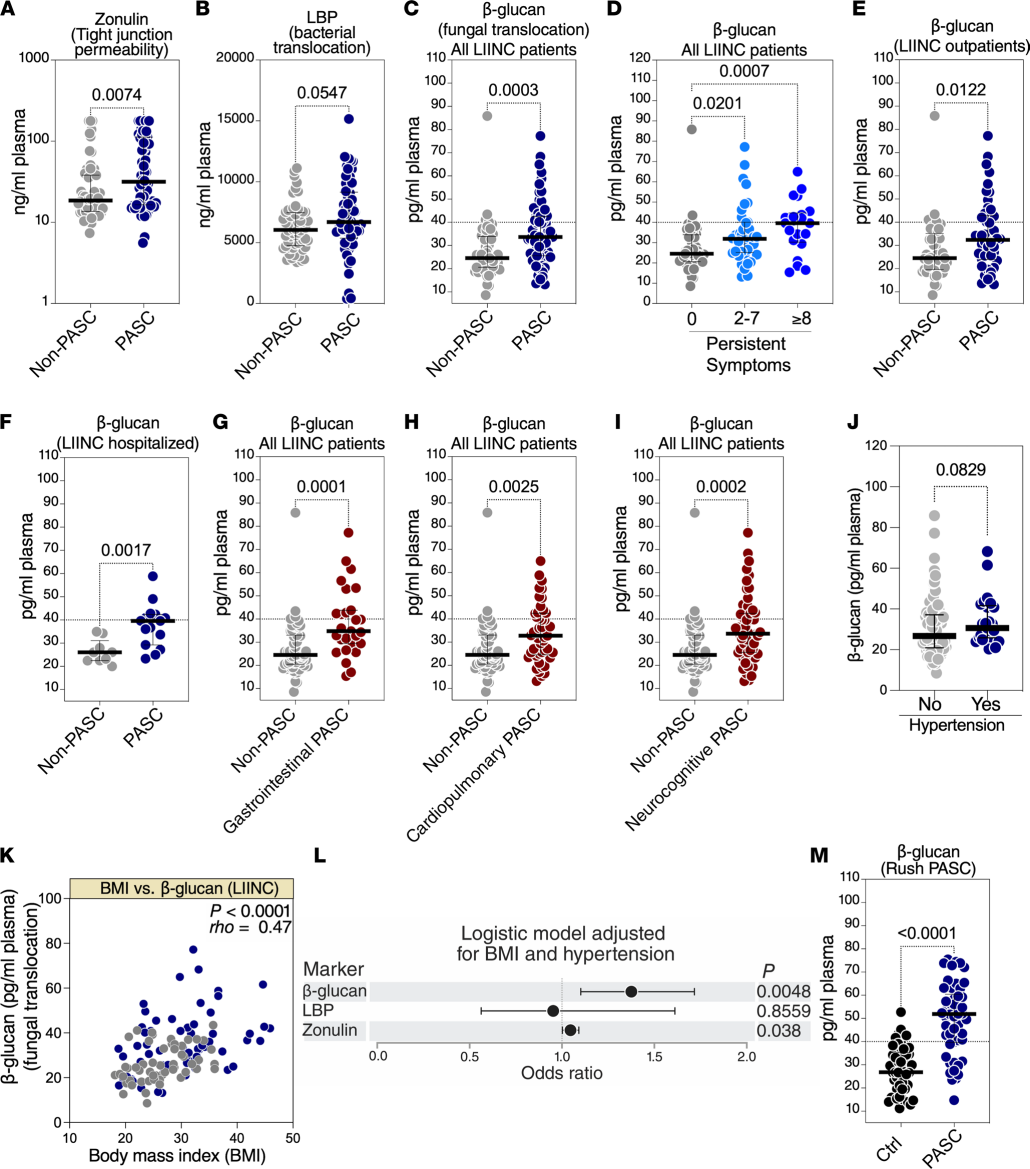
PASC is associated with elevated levels of plasma markers of fungal translocation. (**A**) Levels of zonulin in the plasma of 117 individuals from the UCSF LIINC cohort. Median and IQR are displayed. (**B**) Levels of LBP in the plasma. Median and IQR are displayed. (**C**–**F**) Plasma levels of β-glucan. Levels of β-glucan are higher in PASC compared with non-PASC when analyzing all individuals (**C**), dividing the PASC group into individuals with 2–7 symptoms (*n* = 40) or ≥8 symptoms (*n* = 21) (**D**), analyzing only samples from individuals who were cared for as outpatients during their acute COVID-19 illness (**E**), or analyzing only samples from individuals hospitalized during their acute COVID-19 illness (**F**). Mann-Whitney *U* tests. Median and IQR are displayed. (**G**–**I**) PASC was divided to 3 PASC phenotypes based on clinical symptom clusters, defined as having at least 1 symptom in the cluster — GI (nausea, diarrhea, loss of appetite, abdominal pain, vomiting), cardiopulmonary (cough, dyspnea, chest pain, palpitations), and neurocognitive (headache, concentration problems, dizziness, balance problems, neuropathy, vision problems). Levels of β-glucan were higher in individuals experiencing each of the 3 PASC symptom clusters compared with non-PASC. Mann-Whitney *U* tests. Median and IQR are displayed. (**J**) Mann-Whitney *U* comparison of the levels of β-glucan in individuals with or without a history of hypertension. Median and IQR are displayed. (**K**) Spearman’s rank correlation between BMI and the plasma levels of β-glucan. (**L**) A multivariate logistic model showing that the higher levels of β-glucan and zonulin (OR per 5-unit increase) can differentiate PASC from non-PASC within the UCSF LIINC cohort after adjusting for both BMI and hypertension. (**M**) Levels of β-glucan in individuals experiencing PASC in an independent validation cohort (Rush PASC cohort) compared with SARS-CoV-2–negative controls (matched for age and sex); Mann-Whitney *U* test. Median and IQR are displayed.

We first examined levels of tight junction permeability (measured as plasma levels of zonulin) in the plasma of the 117 volunteers from the UCSF LIINC cohort. Zonulin is an established physiological mediator of tight junction permeability in the digestive tract, where higher levels of zonulin drive an increase in fungal and bacterial translocation (53, 64, 65). We found that PASC was associated with an increase in the plasma levels of zonulin compared with non-PASC (Figure 2A). We next examined levels of bacterial translocation (measured as LPS binding protein, LBP); these levels were not significantly different between the 2 groups, though a trend of higher levels of LBP in individuals experiencing PASC (than in non-PASC) was observed (Figure 2B). We next examined levels of fungal translocation (measured as β-glucan, a fungal wall polysaccharide). We observed higher levels of β-glucan in the plasma of volunteers with PASC than of non-PASC volunteers (in a manner linked to the number of persistent symptoms and regardless of whether volunteers had been outpatients or hospitalized during their acute COVID-19; Figure 2, C–F). Recently, it was shown that plasma β-glucan levels ≥ 40 pg/mL are clinically significant and associated with higher inflammation and worse survival in patients with acute respiratory failure (66). In the UCSF LIINC cohort, 33% of volunteers experiencing PASC had β-glucan levels ≥ 40 pg/mL, whereas only 7.1% of non-PASC volunteers had β-glucan ≥ 40 pg/mL. We also divided the PASC into 3 PASC phenotypes based on clinical symptom clusters, defined as having at least 1 symptom in the cluster **—** GI (nausea, diarrhea, loss of appetite, abdominal pain, vomiting), cardiopulmonary (cough, dyspnea, chest pain, palpitations), and neurocognitive (headache, concentration problems, dizziness, balance problems, neuropathy, vision problems). Levels of β-glucan were higher in individuals experiencing each of the 3 PASC symptom clusters compared with individuals who were not experiencing PASC (Figure 2, G–I). Furthermore, we investigated individuals experiencing each symptom separately and found that β-glucan levels were higher in individuals with certain symptoms, such as GI symptoms (nausea and diarrhea), vision problems, sleep problems, neuropathy, and pain (Supplemental Figure 1; supplemental material available online with this article; https://doi.org/10.1172/jci.insight.160989DS1). Last, we examined levels of soluble CD14 (sCD14) and soluble CD163 (sCD163) (markers of microbe-mediated myeloid inflammation) but found no significant difference between the 2 groups (*P* > 0.05).

Given that we identified BMI and hypertension as potential confounders of the PASC phenotype in the UCSF LIINC cohort, we examined whether levels of plasma β-glucan correlated with BMI and/or hypertension. We found that individuals with hypertension tended to have higher levels of β-glucan (Figure 2J). We also found that higher BMI correlated with higher levels of plasma β-glucan (Figure 2K). This is consistent with recent reports suggesting that obesity is associated with changes in the intestinal mycobiome and with increases in levels of plasma β-glucan (67, 68). As such, we used a multivariate logistic regression model adjusting for BMI and hypertension and found that higher levels of β-glucan (OR 1.4 per every 5-unit increase; *P* = 0.0048) and zonulin (OR 1.05 per every 5-unit increase; *P* = 0.038) remained associated with the PASC phenotype independently from BMI and/or hypertension (Figure 2L). The high levels of fungal translocation during PASC were confirmed using PASC samples from the Rush PASC cohort; these PASC samples were compared with samples from age- and sex-matched SARS-CoV-2–negative controls (Figure 2M). In the Rush PASC cohort, the majority (74%) of individuals with PASC had β-glucan levels ≥ 40 pg/mL, whereas only 12% of SARS-CoV-2–negative controls had β-glucan levels ≥ 40 pg/mL (Figure 2M). Together these data suggest that PASC is associated with elevated levels of markers of tight junction permeability (zonulin) and fungal translocation (β-glucan) to the blood.

### Plasma β-glucan levels associate with markers of inflammation during PASC.

**Figure 3:**
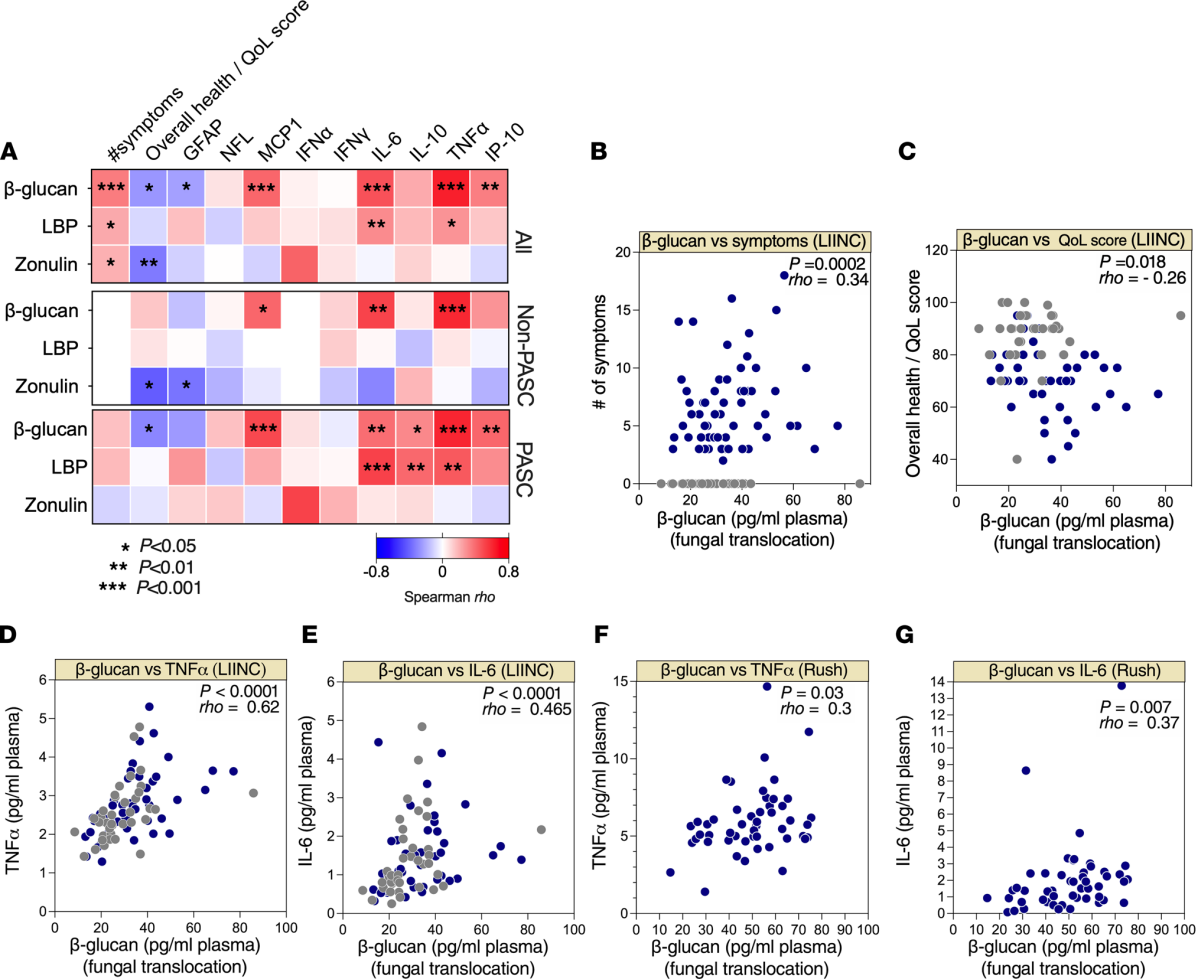
Fungal translocation correlates with inflammation during PASC. (**A**) Three correlation heatmaps showing associations between β-glucan, LBP, or zonulin (in rows) and the number of symptoms during PASC, overall health/quality of life (QoL) score, and plasma levels of several inflammatory markers (in columns) measured in all (*n* = 117; top), non-PASC (*n* = 56; middle), and PASC (*n* = 61; bottom) groups from the UCSF LIINC cohort. The color of the square represents the strength of the Spearman’s rank correlation, where blue shades represent negative correlations and red shades represent positive correlations. *P < 0.05; **P < 0.01; ***P < 0.001. GFAP, glial fibrillary acidic protein; NFL, neurofilament; MCP1, monocyte chemoattractant protein 1; IP-10, IFN-γ–inducible protein of 10 kDa. (**B**–**G**) Examples of the correlations between β-glucan and number of symptoms (UCSF LIINC cohort) (**B**), β-glucan and overall health/QoL score (UCSF LIINC cohort) (**C**), β-glucan and TNF-α or IL-6 (UCSF LIINC cohort) (**D** and **E**), and β-glucan and TNF-α or IL-6 (Rush PASC cohort) (**F** and **G**). Spearman’s rank correlation tests were used for statistical analysis. Blue = PASC, and gray = non-PASC in **B**–**E**.

It is well established that β-glucan can directly induce inflammation following its binding to Dectin-1 on macrophages, monocytes, and dendritic cells. This activates the NF-κB pathway and induces the secretion of proinflammatory cytokines (69–71). In addition, the exposure of myeloid cells to β-glucan can modulate several cellular metabolic (including glutathione metabolism) and epigenetic pathways that induce cytokine production (72). We, therefore, tested whether β-glucan levels correlated with markers of inflammation, as well as number of symptoms and overall health/QoL score (Figure 3A). We found a positive correlation between β-glucan levels of inflammatory markers, including TNF-α, IL-6, and IP-10 (Figure 3, A, D, and E). Levels of β-glucan also associated with a higher number of symptoms (Figure 3, A and B) and a lower overall health/QoL score (Figure 3, A and C). The positive correlations between levels of β-glucan and higher IL-6 and TNF-α were confirmed in the PASC samples from the Rush PASC cohort (Figure 3, F and G). These data suggest a potential link between fungal translocation and inflammation in individuals with PASC.

### Plasma β-glucans from patients with PASC activate the NF-κB pathway.

**Figure 4:**
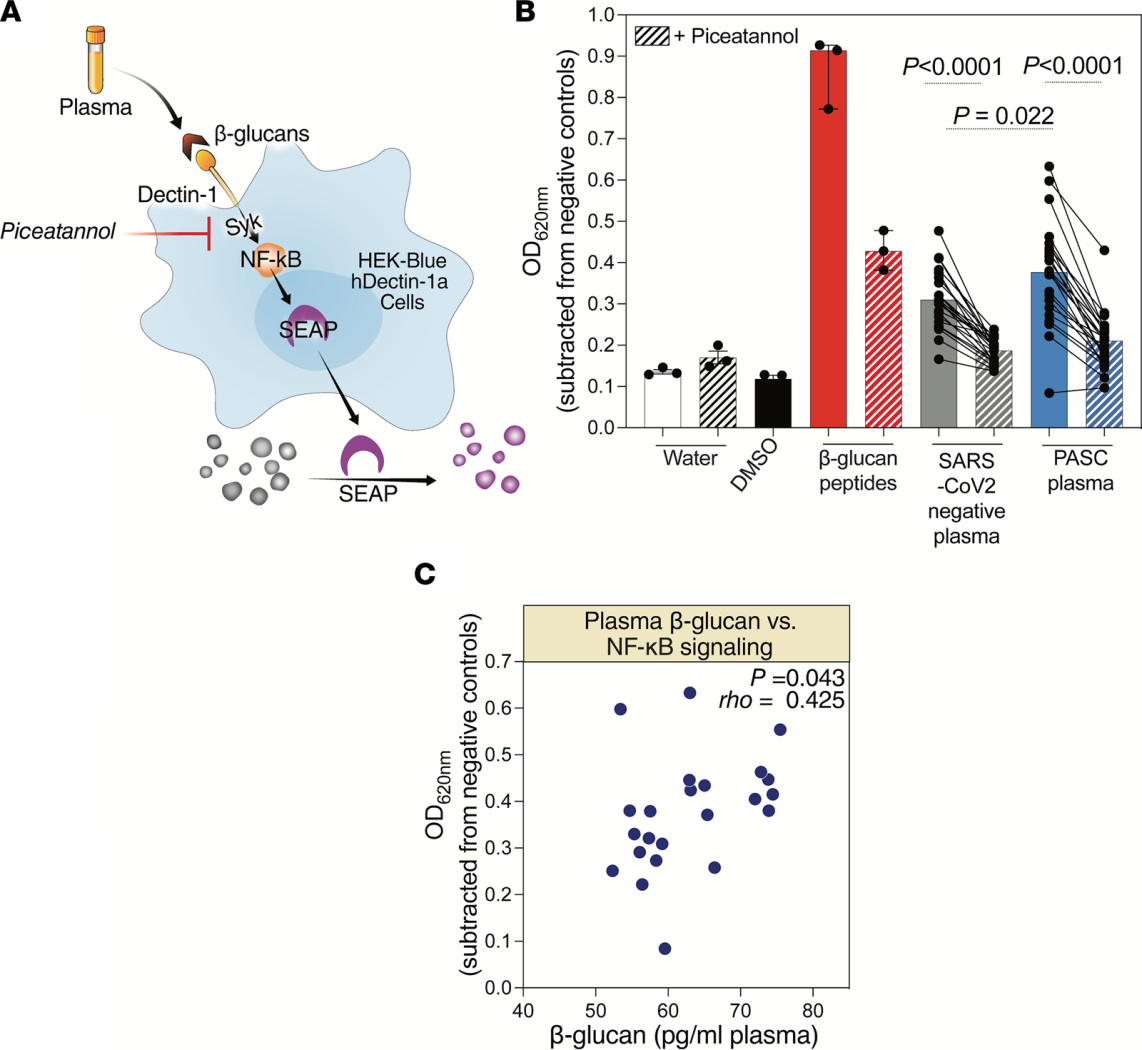
Plasma from individuals with PASC induces NF-κB signaling, which is dampened by the spleen tyrosine kinase inhibitor piceatannol. (**A**) A schematic of the Dectin-1 receptor reporter cell line. This cell line stably expresses the Dectin-1 receptor and an NF-κB reporter linked to SEAP so that Dectin-1 receptor stimulation by β-glucan can be measured by quantifying SEAP activity. Syk, spleen tyrosine kinase. (**B**) The Dectin-1 receptor reporter cells were treated with plasma from individuals with PASC or SARS-CoV-2–negative controls in the presence or absence of the Syk inhibitor piceatannol. NF-κB signaling was detected (OD_620 nm_) 24 hours later. The comparison between the SARS-CoV-2–negative controls and PASC samples without piceatannol was performed using Mann-Whitney *U* test, and the comparisons between the conditions with and without piceatannol were performed using Wilcoxon’s signed rank tests. (**C**) Spearman’s rank correlation between levels of β-glucan in the plasma during PASC and NF-κB signaling induced by PASC plasma, using the Dectin-1 receptor reporter system.

The data described thus far suggest that PASC is associated with high plasma levels of β-glucan in a manner linked to higher inflammation. Although modest compared with levels observed during invasive fungal infections, the levels of plasma β-glucan we observed in 2 cohorts of PASC (Figures 2 and 3) may be clinically significant and with a potential to exacerbate a proinflammatory state, as suggested by a recent study (66). In that study (not focused on COVID-19), β-glucan levels in plasma of 40 pg/mL or greater were associated with higher inflammation, fewer ventilator-free days, and worse survival in patients with acute respiratory failure (66). It is known that β-glucans induce inflammation by activating the NF-κB pathway following binding to the Dectin-1 receptor (69–71). We, therefore, examined whether there was a mechanistic link between the levels of β-glucans in the plasma of individuals with PASC and the Dectin-1–dependent activation of the NF-κB pathway. For these experiments, we used a Dectin-1 receptor reporter cell line to measure β-glucan/Dectin-1–dependent NF-κB signaling. This cell line stably expresses the Dectin-1 receptor and an NF-κB reporter linked to secreted embryonic alkaline phosphatase (SEAP) so that Dectin-1 receptor stimulation by β-glucan can be measured by quantifying SEAP activity (Figure 4A).

We treated the Dectin-1 receptor reporter cells with 20 μL of plasma from volunteers with PASC (a subset of the Rush PASC cohort) or 20 μL of plasma from SARS-CoV-2–negative controls. The plasma from the PASC group induced significantly higher levels of NF-κB activation (in a Dectin-1– and β-glucan–dependent manner) compared with the plasma from SARS-CoV-2–negative controls (Figure 4B). This β-glucan/Dectin-1–dependent NF-κB signaling was significantly abrogated by the addition of a selective inhibitor to Syk signaling (piceatannol) (Figure 4B). Finally, levels of β-glucan in the plasma of those with PASC correlated with a higher ability of these plasma samples to induce β-glucan/Dectin-1–dependent NF-κB signaling using this reporter system (Figure 4C). These data suggest that the levels of β-glucan in the plasma during PASC are capable of inducing immune activation in a manner linked to the activation of the Dectin-1/Syk/NF-κB signaling pathway. Together, these observations suggest a potential mechanism by which fungal translocation may contribute to inflammation during PASC. Importantly, this inflammation can be inhibited using inhibitors to Dectin-1/Syk signaling, providing a potential approach to mitigate PASC.

### PASC is associated with elevated levels of host metabolic agonists of NMDA receptors with established neurotoxic properties.

**Figure 5:**
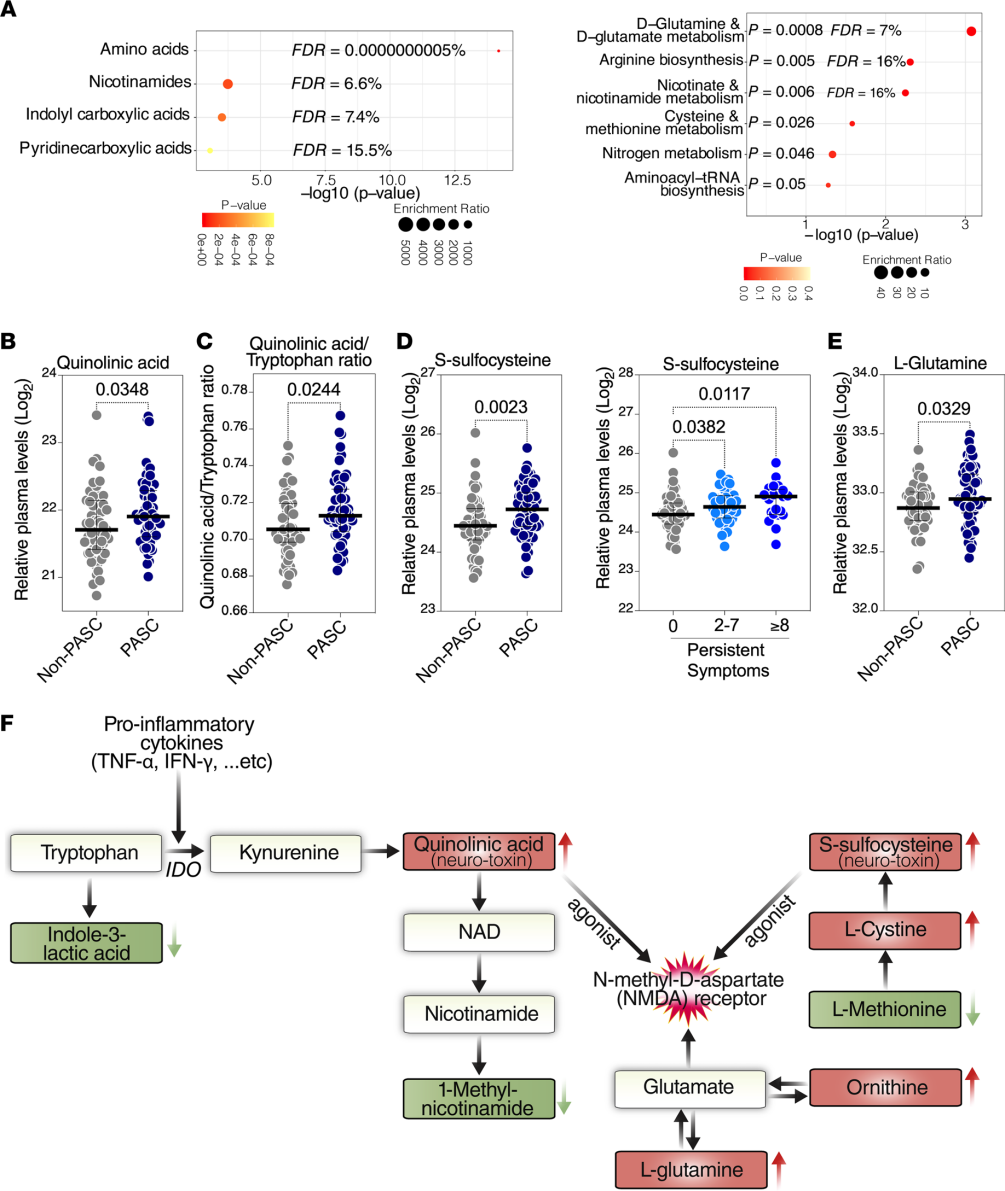
PASC is associated with elevated levels of host metabolic agonists of NMDA receptors with established neurotoxic properties. (**A**) Unbiased enrichment analyses of the 12 plasma metabolites whose levels differed between PASC and non-PASC groups within the UCSF LIINC cohort. Analysis was performed using MetaboAnalyst 5.0 (http://www.metaboanalyst.ca/). Left image: enrichment of certain classes of metabolites. Right image: enrichment of certain metabolic pathways using the Kyoto Encyclopedia of Genes and Genomes database. (**B**–**E**) Mann-Whitney *U* comparisons of the plasma levels of quinolinic acid (**B**), quinolinic acid/tryptophan (Q/T) ratio (**C**), S-sulfocysteine (**D**), or l-glutamine (**E**) in PASC and non-PASC groups from the UCSF LIINC cohort. Median and IQR are displayed. (**F**) A model depicting 8 plasma metabolites whose levels differed between PASC from non-PASC groups (red indicates higher in PASC than non-PASC, and green indicates lower in PASC than non-PASC) within the UCSF LIINC cohort and their relationship to both the tryptophan catabolism pathway and the activation of the NMDA receptors. IDO, indoleamine 2,3-dioxygenase.

Microbial translocation–mediated inflammation can not only impact biological functions directly, but also impact them indirectly by modulating the circulating levels of metabolites derived from interactions between gut microbiota and the host. Many plasma metabolites are biologically active molecules able to regulate cellular processes and immunological functions (73). For example, inflammation-mediated tryptophan catabolism has been associated with the development of several aging- and inflammation-associated diseases during HIV infection (74–78). Severe acute COVID-19 has been associated with a disruption in the levels of several host metabolites such as the metabolites involved in the tryptophan catabolism pathway and S-sulfocysteine (55, 79). We therefore performed an untargeted metabolic analysis (using liquid chromatography-tandem mass spectrometry, LC-MS/MS) on the plasma samples from the UCSF LIINC cohort. Within the 117 plasma samples, we identified 169 polar metabolites. We observed a significant (with nominal *P* < 0.05) difference between PASC and non-PASC groups in 12 of these metabolites (6 were higher in the PASC compared with non-PASC group, and 6 were lower in the PASC compared with non-PASC group: Supplemental Table 1). Untargeted metabolite enrichment analysis of these 12 PASC-associated metabolites showed an enrichment of amino acids and certain amino acid–related metabolic pathways (Figure 5A).

Among the differences between PASC and non-PASC groups were higher levels of quinolinic acid, a downstream product of the tryptophan catabolism pathway, in those with PASC compared with the non-PASC group (Figure 5B). Tryptophan catabolism is commonly indicated by 2 ratios, the kynurenine-to-tryptophan (K/T) ratio and the quinolinic acid–to-tryptophan (Q/T) ratio (80). Although we did not observe a statistically significant difference in the K/T ratio between PASC and non-PASC groups, the Q/T ratio was higher in those with PASC compared with the non-PASC group (Figure 5C). Higher levels of quinolinic acid and a higher Q/T ratio have been associated with adverse disease outcomes during chronic HIV infection (74, 80). Quinolinic acid is an established neurotoxin and NMDA receptor agonist (81, 82). Interestingly, other metabolites that activate NMDA receptors were elevated in the plasma of those with PASC, such as S-sulfocysteine (83) (Figure 5D) and l-glutamine (Figure 5E). Indeed, several of the 12 metabolites that differed between those with and without PASC are involved in pathways related to the activation of NMDA receptors, as shown in the diagram in Figure 5F. Consistent with the neurotoxic ability of quinolinic acid and S-sulfocysteine, higher Q/T and K/T ratios were associated with neuropathy during PASC (Supplemental Figure 2), and higher levels of S-sulfocysteine were associated with neurocognitive PASC (and the other 2 PASC phenotypes; Supplemental Figure 3). S-sulfocysteine levels were also associated with particular neurological symptoms during PASC, such as vision problems, fatigue, headache, and dizziness (Supplemental Figure 4). Together, these data indicate that a metabolic signature associated with PASC is compatible with increased tryptophan catabolism and accumulation of metabolites with neurotoxic properties, conferred by their ability to activate NMDA receptors.

### Plasma metabolomic markers of PASC are associated with higher inflammation and lower overall health.

**Figure 6:**
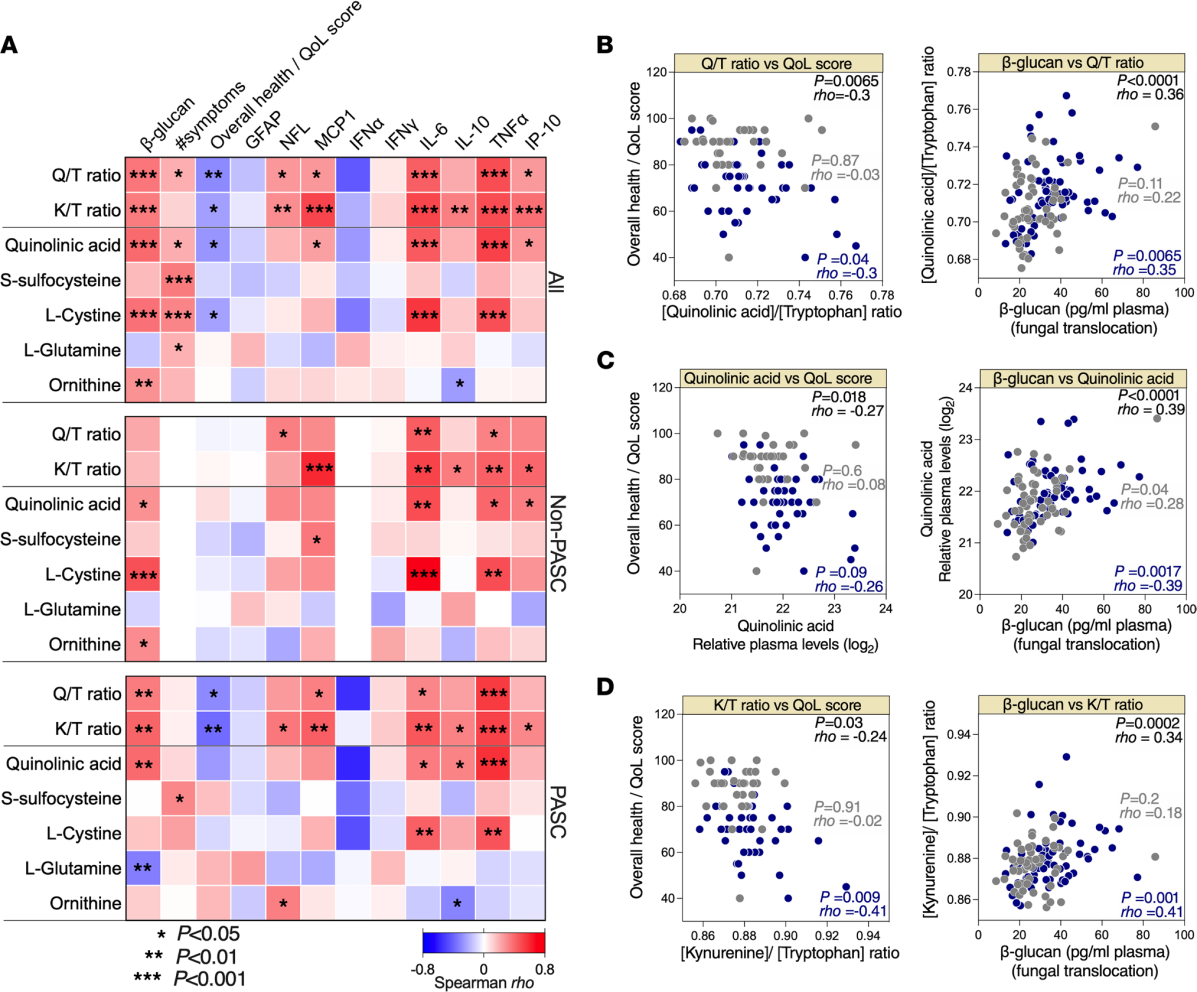
Levels of plasma host metabolites correlate with inflammation during PASC. (**A**) Three correlation heatmaps showing associations between Q/T ratio, K/T ratio, and levels of selected metabolites (in rows) to levels of plasma β-glucan, the number of symptoms during PASC, overall health/QoL score, and plasma levels of several inflammatory markers (in columns) measured in all (*n* = 117; top), non-PASC (*n* = 56; middle), and PASC (*n* = 61; bottom) individuals from the UCSF LIINC cohort. The color of the square represents the strength of the Spearman’s rank correlation, with blue shades representing negative correlations and red shades representing positive correlations. *P < 0.05; **P < 0.01; ***P < 0.001. (**B**) Examples of the correlations between Q/T ratio and overall health/QoL score or β-glucan. (**C**) Examples of the correlations between quinolinic acid and overall health/QoL score or β-glucan. (**D**) Examples of the correlations between K/T ratio and overall health/QoL score or β-glucan. Spearman’s rank correlation tests were used for statistical analysis. Blue = PASC, and gray = non-PASC in **B**–**D**.

As bioactive molecules, plasma metabolites influence cellular processes and immunological responses. Therefore, we asked whether any of the 12 dysregulated plasma metabolites, as well as Q/T and K/T ratios (as markers of tryptophan catabolism), associated with levels of β-glucan, number of symptoms, overall health score, or plasma markers of inflammation (Figure 6A shows heatmaps focusing on the correlations between Q/T ratio, K/T ratio, and 5 elevated metabolites; a complete list of correlations is shown in Supplemental Table 2). The most significant associations were between Q/T ratio, quinolinic acid, or K/T ratio and lower overall health score (only in the PASC group but not in the non-PASC group; Figure 6, A–D). In addition, levels of quinolinic acid (and other elevated metabolites) and Q/T and K/T ratios correlated with higher levels of markers of inflammation and higher levels of β-glucan (Figure 6, A–D), mainly during PASC. Notably, the positive correlations between β-glucan levels and markers of the tryptophan catabolism pathway (such as the K/T ratio) were also recently observed during HIV infection (84). Together, these data further support potential links between disrupted metabolic activities, especially those related to tryptophan catabolism and NMDA receptor activation, and both inflammation and disease severity during PASC.

## Discussion

**Figure 7:**
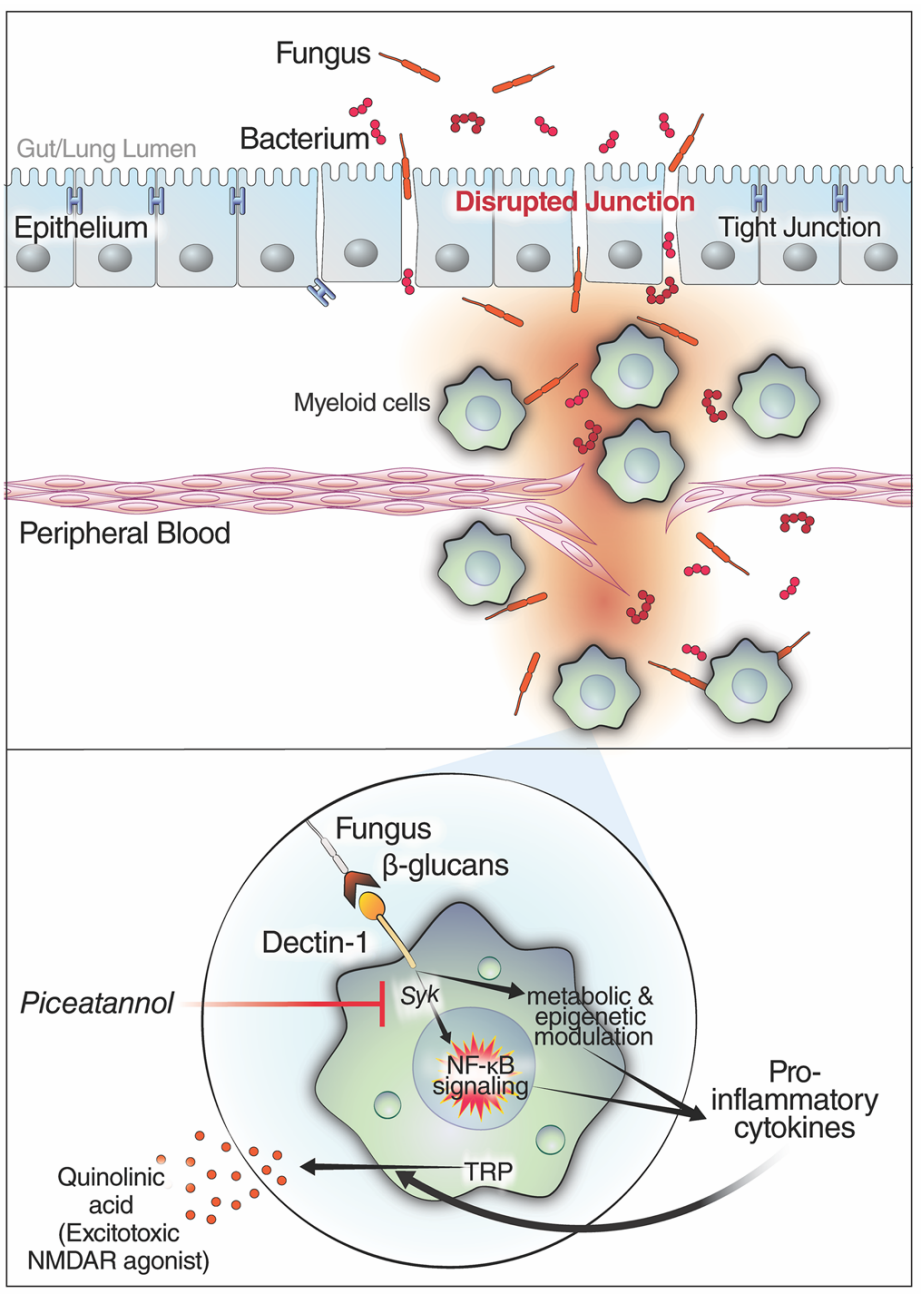
Model of how fungal translocation may contribute to inflammation during PASC. Top: Our data suggest that during PASC there are elevated levels of fungal translocation from the gut and/or lung to the blood (possibly driven by increases in the tight junction permeability driver, zonulin). Bottom: The translocated β-glucans (fungal cell wall polysaccharides) bind to Dectin-1 on myeloid cells to activate the cellular inflammasome via the NF-κB pathway. Blocking the Syk pathway (using piceatannol) prevents the β-glucan–mediated myeloid inflammation in vitro and may prevent it during PASC. Finally, the inflammation (proinflammatory cytokines) can activate the tryptophan (TRP) catabolism pathway, and cause other metabolic dysregulations, to induce levels of host metabolic agonists of the NMDA receptors (NMDARs; such as quinolinic acid) with established neurotoxic properties.

Identifying the potential mechanisms underlying the sustained elevated levels of immune activation and inflammation during PASC is a critical step toward developing tools to prevent or decrease the severity of this syndrome. Our data support a model where a disturbance in tight junction permeability (in the gut and/or lung) allows fungal translocation to the blood. The fungal β-glucan can then induce the production of proinflammatory cytokines by binding to its receptor (Dectin-1) on the surface of myeloid cells and activating the Syk/NF-κB pathway. This activation can be inhibited with the Syk inhibitor piceatannol. It has also been shown that the exposure of myeloid cells to β-glucan modulates several cellular metabolic and epigenetic pathways that can induce cytokine production (72). Our data also suggest that elevation of specific microbiome-associated and inflammation-associated metabolic pathways may contribute to PASC, in particular, pathways with neurotoxic properties due to activation of NMDA receptors (Figure 7). It is unlikely that the elevated levels of fungal translocation and neurotoxic host metabolites are the primary triggers of PASC, as this complex clinical syndrome likely results from the disruption of multiple and probably distinct pathophysiological pathways. However, the robust literature indicating that fungal translocation fuels inflammation and disease severity during long-term complications of other viral infections, such as HIV (28, 31), supports and is consistent with our findings and suggests that fungal translocation may be one of the mechanisms contributing to inflammation during PASC.

Our in vitro experiments suggest that the levels of β-glucan in the plasma of individuals experiencing PASC are sufficient to induce NF-κB activation in vitro and show that this activation is greater in those with PASC than in SARS-CoV-2–negative controls. As noted above, β-glucan is a biomarker of microbial translocation during chronic viral infections, such as HIV infection, and its levels correlate with inflammation, immune suppression, and the development of HIV-associated comorbidities (50, 52, 67, 84–86). It can also directly induce inflammation following its binding to Dectin-1 (69–71). Thus, these data suggest a mechanism, Dectin-1/Syk/NF-κB signaling, by which the increased fungal translocation during PASC may contribute to the observed sustained elevated levels of immune activation and inflammation. However, a deeper mechanistic analysis will be needed to identify the degree to which this NF-κB activation contributes to inflammation during PASC. Further analyses could also investigate the possibility that myeloid cells (and other immune cells expressing Dectin-1) from individuals with PASC are less resistant to β-glucan stimulation than cells from individuals without PASC. Together, this deeper analysis could shed light on the causative versus consequential links between fungal translocation, inflammation, and PASC.

Our results support the development of novel strategies to prevent or treat PASC, such as microbial interaction–targeted therapeutics (such as probiotics or metabolites) and/or selective small molecules. For example, small molecules that enhance epithelial barrier integrity or reduce the detrimental effects of fungal translocation are available, including the zonulin receptor antagonist AT1001 (larazotide acetate); this antagonist decreased the severity and incidence of several inflammation-associated diseases in preclinical and clinical studies (87–89) and successfully treated a 17-month-old boy with SARS-CoV-2–associated multisystem inflammatory syndrome in children who did not respond to antiinflammatory therapies (90). Also available are the Dectin-1 antagonist, laminarin, which has been used safely and successfully in mouse models of ischemic stroke (91) and colitis (92), and the Syk signaling inhibitor piceatannol, which has been used to treat a mouse model of ischemic stroke (91). Our in vitro data suggest that piceatannol may abrogate β-glucan–mediated inflammation during PASC. These molecules can form a foundation for designing strategies — to be tested preclinically as soon as preclinical models of PASC are available — to prevent PASC and its long-term complications in individuals recovering from SARS-CoV-2 and/or other similar post-acute infection syndromes.

Aging, several aging-associated diseases, and even other chronic viral infections, have been associated with a breakdown of homeostasis between the gut and its microbiome (93–95). For example, aging itself changes the composition of the gut microbiota (96–100), leading to microbial translocation, which triggers chronic inflammation (101–103). Aging-associated diseases such as cancer, diabetes, and Alzheimer’s disease are associated with specific gut microbial signatures (104–114). Chronic HIV infection is associated with a state of gut microbial translocation, which is thought to be a major cause of inflammation (115–123). Even with antiretroviral therapy, the damage to the epithelial barrier caused by HIV is never fully repaired, allowing microbial translocation and inflammation to continue (124–126). Our data raise the question of whether the preexisting state of microbial translocation and chronic inflammation during these conditions might make individuals living with them more prone to PASC. In this analysis, we included only a small number of HIV-infected individuals (we analyzed a subset of the UCSF LIINC cohort); however, a recent study focused on LIINC participants with HIV suggested that HIV infection is indeed a risk factor for developing PASC (127). Whether the preexisting microbial translocation and chronic inflammation during HIV infection (115–126), after surviving Ebola disease (128), and/or during other aging-related conditions (104–114) contribute to PASC warrants further investigation using longitudinal samples before and after acute COVID-19.

Our metabolic analysis suggests that PASC is associated with elevated levels of several metabolites in a manner linked to fungal translocation. Our in vivo analyses do not unequivocally demonstrate a causal relationship between microbial translocation and host metabolic dysregulation during PASC. However, the robust literature indicating a link between microbial dysbiosis/translocation and host metabolic dysregulation (for example, an increase in tryptophan catabolism) in a manner that can fuel inflammation and disease severity is consistent with our findings (129, 130). Nevertheless, future mechanistic studies will be needed to demonstrate a causal relationship.

Our data suggest that PASC is associated with elevated levels of several metabolites with known neurotoxic properties that are linked to activation of NMDA receptors, such as quinolinic acid and S-sulfocysteine. The overactivation of NMDA receptors can lead to excitotoxicity and has been associated with the development of several neurodegenerative disorders, including epilepsy and Parkinson’s, Alzheimer’s, and Huntington’s diseases (131–135). Whether an overactivation of NMDA receptors due to dysregulation of host metabolites contributes to neuropathology during PASC warrants investigation. A demonstration that NMDA receptor agonists contribute to PASC symptoms could have several clinical implications. For example, NMDA receptor antagonists (such as memantine) have been used to block excessive, excitotoxic activity resulting from the overactivation of the NMDA receptors during Alzheimer’s disease (132–136) and have been evaluated for treating other neurodegenerative disorders (137–139). Whether memantine or other NMDA receptor antagonists can be used to prevent or treat PASC-associated neurological manifestations could then be explored.

This study has several limitations. It is not clear why markers of fungal translocation, but not bacterial translocation, correlated with PASC in our study. A possible explanation is a difference in the kinetics of these markers in blood. It was recently shown that β-glucan levels in the blood are more stable and less sensitive to time of sample collection, relative to food uptake, than are LPS levels (140). Other explanations could include differences in assay sensitivity, specificity, and/or criteria (for example, some soluble markers can be more sensitive than other markers to circadian rhythm). Also, the source of β-glucan in the plasma is not clear. Fungal translocation during illness can occur from both the gut and the lung (66). Future studies examining the contribution of the gut microbiome (both bacterial and fungal; using stool samples and intestinal biopsies) and lung microbiome (both bacterial and fungal; using sputum and/or bronchoalveolar lavage) will be needed to determine the source and potential contribution of both fungal and bacterial translocation to PASC. It will also be important to examine fungal translocation and host metabolites in longitudinal samples (from different body fluids, including cerebrospinal fluid) to determine whether, and for how long, elevated levels of microbial translocation persist after acute COVID-19. Another possible explanation of the persistent dysregulation is that the intestinal barrier’s resilience to common intestinal disruptors (such as excessive alcohol or oxidative stress) is lower during PASC, making these individuals more vulnerable to frequent, common disruptors, which then can lead to the translocation of microbes that cause systemic inflammation. Analyzing the impact of the potential fungal translocation during PASC on immune cell activation (such as the coexpression of CD38 and HLA-DR on T cells), other markers of systemic inflammation and immune dysfunction during COVID-19 (such as GDF-15 and galectins, refs. 55, 141), and the expression of β-glucan receptors (such as Dectin-1 on monocytes and NKp30 on NK cells), in the intestines and systemically, will be also needed in future studies. Finally, correcting for additional potential confounders will require validating our results in larger independent cohorts from varying geographic settings and demographic groups. Despite these caveats, this study, which is exploratory in nature, sheds light on the potential role of microbial translocation and dysregulation of host metabolic pathways related to NMDA receptor activation in the pathophysiology of PASC. By understanding these potential underpinnings of PASC, this work may serve to identify biomarkers for PASC risk stratification and build a foundation for developing strategies to prevent or reduce the severity of inflammation during PASC.

## Methods

### Study cohorts.

Primary analyses were performed using cross-sectional plasma samples from 117 individuals with prior nucleic acid–confirmed SARS-CoV-2 infection (a subset of the UCSF LIINC cohort, described in detail elsewhere; ref. 19) collected 90–160 days after the first positive SARS-CoV-2 quantitative PCR result; prior work has not identified persistent virus in saliva of these individuals at the time of sampling (142). These participants were divided into 2 groups based on responses to a standardized symptom assessment at the time of plasma collection: 56 individuals with no COVID-19–attributed symptoms (non-PASC) and 61 with at least 2 COVID-19–attributed symptoms (PASC; Table 1); individuals reporting a single symptom were not included. Validation analyses and in vitro experiments were performed using cross-sectional plasma samples from 50 individuals with COVID-19 experiencing PASC symptoms 3–4 months after acute COVID-19 (a subset of the Rush PASC cohort) and cross-sectional plasma samples from 50 SARS-CoV-2–negative controls (matched for age and sex to the Rush PASC samples; Table 1).

### Symptoms and QoL score evaluation.

Participants in the LIINC study underwent clinical assessment at the time of biospecimen collection. Volunteers completed an interviewer-administered questionnaire querying the presence of 32 possible COVID-19–attributed symptoms, QoL, and overall health status. The questionnaire was derived from several validated instruments (143, 144) as well as the US CDC list of COVID-19 symptoms. Importantly, a symptom had to be described as new or worsened since the diagnosis of SARS-CoV-2 infection to be recorded as “present”; symptoms that existed prior to SARS-CoV-2 infection or were unchanged following infection were not counted. The utility of this instrument in measuring participants longitudinally has been described (19).

### Measurement of plasma markers of tight junction permeability and microbial translocation.

Plasma levels of sCD14, sCD163, and LBP were quantified using DuoSet ELISA kits (R&D Systems, Bio-Techne; catalog DY383-05, DY1607-05, and DY870-05, respectively). The plasma level of zonulin was measured using an ELISA kit from MyBioSource (catalog MBS167049). Detection of β-d-glucan in plasma was performed using Limulus Amebocyte Lysate assay (Glucatell kit, Associates of Cape Cod; catalog GT003).

### Measurement of plasma markers of inflammation.

A targeted panel of markers of inflammation in plasma were measured using the Simoa HD-X platform. These markers were selected based on their importance in acute SARS-CoV-2 infection and included Cytokine 3-PlexA (IL-6, IL-10, TNF-α), IFN-γ, IP-10, and MCP-1. Levels of these markers in a subset of LIINC participants have been previously reported (22, 24). The IL-6 and TNF-α measurements of the PASC samples from the Rush PASC cohorts were performed using a customized U-PLEX multiplex assay (Meso Scale Diagnostic catalog K15067L-2).

### Untargeted measurement of plasma metabolites.

Metabolomics analysis was performed as described previously (145, 146). Briefly, polar metabolites were extracted with 80% methanol. A quality control (QC) sample was generated by pooling equal volumes of all samples and was injected periodically during the sample sequence. LC-MS was performed on a Thermo Fisher Scientific Q Exactive HF-X mass spectrometer with HESI II probe and Vanquish Horizon UHPLC system. Hydrophilic interaction liquid chromatography (HILIC) was performed at 0.2 mL/min on a ZIC-pHILIC column (2.1 mm × 150 mm, MilliporeSigma) at 45°C. All samples were analyzed by full MS with polarity switching, and the QC sample was also analyzed by data-dependent MS/MS with separate runs for positive and negative polarities. Raw data were analyzed using Compound Discoverer 3.1 (Thermo Fisher Scientific). Metabolites were identified either by accurate mass and retention time using an in-house database generated from pure standards or by querying the mzCloud database (https://www.mzcloud.org) with MS/MS spectral data and selecting matches with 50 or greater scores. Metabolite quantification used integrated peak areas from full MS runs. These values were corrected based on the periodic QC runs and normalized to the total signal from identified metabolites in each sample.

### Reporter assay for Dectin-1 activation by β-glucan.

HEK-Blue hDectin-1a cells (InvivoGen; catalog hkb-hdect1a) were maintained in growth medium containing DMEM (4.5 g/L glucose), 10% fetal bovine serum, 100 U/mL penicillin, 100 μg/mL streptomycin, 100 μg/mL Normocin (InvivoGen; catalog ant-nr-05), 2 mM l-glutamine, 1 μg/mL puromycin (InvivoGen; catalog ant-pr-1), and 1× HEK-Blue CLR Selection (InvivoGen; catalog hb-csm). This cell line expresses the Dectin-1a isoform and genes involved in the Dectin-1/NF-κB/SEAP signaling pathway. On the assay day, 180 μL/well of cells at a concentration of 2.8 × 10^5^ cells/mL in HEK-Blue Detection media (InvivoGen; catalog hb-det2) were plated in a 96-well tissue culture plate. Plasma samples (20 μL) were added to each well with and without piceatannol (250 nM). The plates were then incubated at 37°C and 5% CO_2_ for 24 hours. Levels of SEAP were monitored and measured spectrophotometrically at 620 nm. As controls, 20 μL of water, piceatannol (250 nM), 2 μL of DMSO (piceatannol solvent), 20 μL of 10 μg/mL β-glucan peptides (InvivoGen; catalog tlrl-bgp), or 20 μL of β-glucan peptides + piceatannol (250 nM) were added in separated wells.

### Statistics.

Mann-Whitney *U* tests were used in the analyses of Figure 1; A–C; Figure 2, A–J and M; Figure 4B (in the comparisons between the SARS-CoV-2–negative controls and PASC samples without piceatannol); and Figure 5, B–E. Fisher’s exact tests were used in the analysis in Figure 1D. Spearman’s rank correlations were used in the analyses in Figure 2K, Figure 4C, and Figures 3 and 6. A multivariate logistic regression model adjusting for BMI and hypertension was used for each marker in the analysis in Figure 2L. Wilcoxon’s signed rank tests were used in the analysis in Figure 4B (in the comparisons between the conditions with and without piceatannol). Kruskal-Wallis tests were used in the analyses in Supplemental Figures 1–3. All statistical analyses were performed in R and Prism 9.0 (GraphPad).

### Study approval.

All research protocols were approved by the institutional review boards at UCSF, Rush University, and The Wistar Institute. All human experimentation was conducted in accordance with the guidelines of the US Department of Health and Human Services and those of the authors’ institutions.

## Author contributions

MAM conceived and designed the study. LBG, JK, KYH, ARG, and HYT carried out the experiments. BCY, AC, JWW, and CJP measured the inflammatory markers. MJP, GK, NFZ, HR, GEM, FB, RAB, JN Moy, RH, SL, JDK, HW, JN Martin, AK, AL, SGD, and TJH screened and selected study participants; supervised all clinical aspects of the study; and collected and interpreted the clinical data. JD and QL analyzed the data. LBG and MAM wrote the manuscript, and all authors edited it.

## Supplementary Material

Supplemental data

Supplemental table 1

## Figures and Tables

**Table 1:**
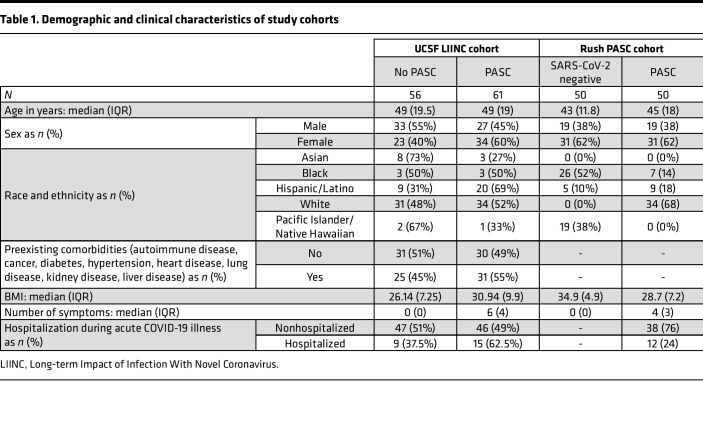
Demographic and clinical characteristics of study cohorts

## Version 1 06/21/2022

In-Press Preview

## Version 2 08/08/2022

Electronic publication
