# Markers of fungal translocation are elevated during post-acute sequelae of SARS-CoV-2 and induce NF-κB signaling

**DOI:** 10.1172/jci.insight.164813

**Published:** 2022-09-22

**Authors:** Leila B. Giron, Michael J. Peluso, Jianyi Ding, Grace Kenny, Netanel F. Zilberstein, Jane Koshy, Kai Ying Hong, Heather Rasmussen, Gregory E. Miller, Faraz Bishehsari, Robert A. Balk, James N. Moy, Rebecca Hoh, Scott Lu, Aaron R. Goldman, Hsin-Yao Tang, Brandon C. Yee, Ahmed Chenna, John W. Winslow, Christos J. Petropoulos, J. Daniel Kelly, Haimanot Wasse, Jeffrey N. Martin, Qin Liu, Ali Keshavarzian, Alan Landay, Steven G. Deeks, Timothy J. Henrich, Mohamed Abdel-Mohsen

Original citation: *JCI Insight*. 2022;7(15):e160989. https://doi.org/10.1172/jci.insight.160989

Citation for this corrigendum: *JCI Insight*. 2022;7(18):e164813. https://doi.org/10.1172/jci.insight.164813

For clarity, the authors have updated Table 1 so that the percentages of each variable in the UCSF LIINC cohort reflect percentages within each group (No PASC and PASC). The correct Table 1 is below. The online HTML and PDF versions of the manuscript have been updated to reflect these changes.

The authors regret the errors. 

## Figures and Tables

**Table 1:**
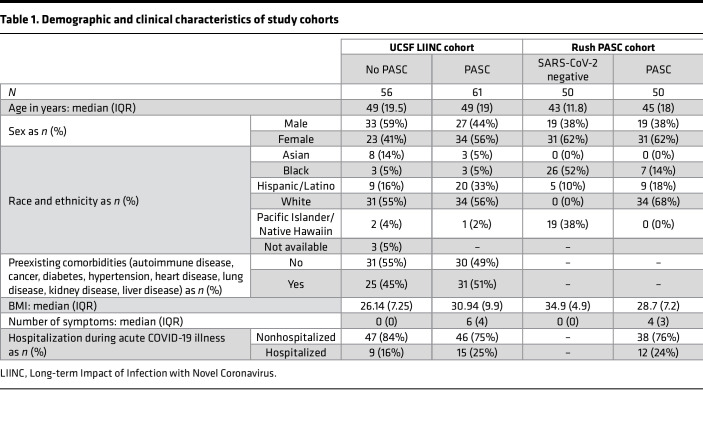
Demographic and clinical characteristics of study cohorts

## Version 1 09/22/2022

Electronic publication

