# Supplementary material for: Markers of fungal translocation are elevated during post-acute sequelae of SARS-CoV-2 and induce NF-κB signaling
Source: JCI Insight. 2022 Aug 8;7(15):e160989. doi: 10.1172/jci.insight.160989 (PMC9462470; doi:10.1172/jci.insight.160989)
Supplement: Supplemental data [file jciinsight-7-160989-s125.pdf]

Supplementary Figure 1

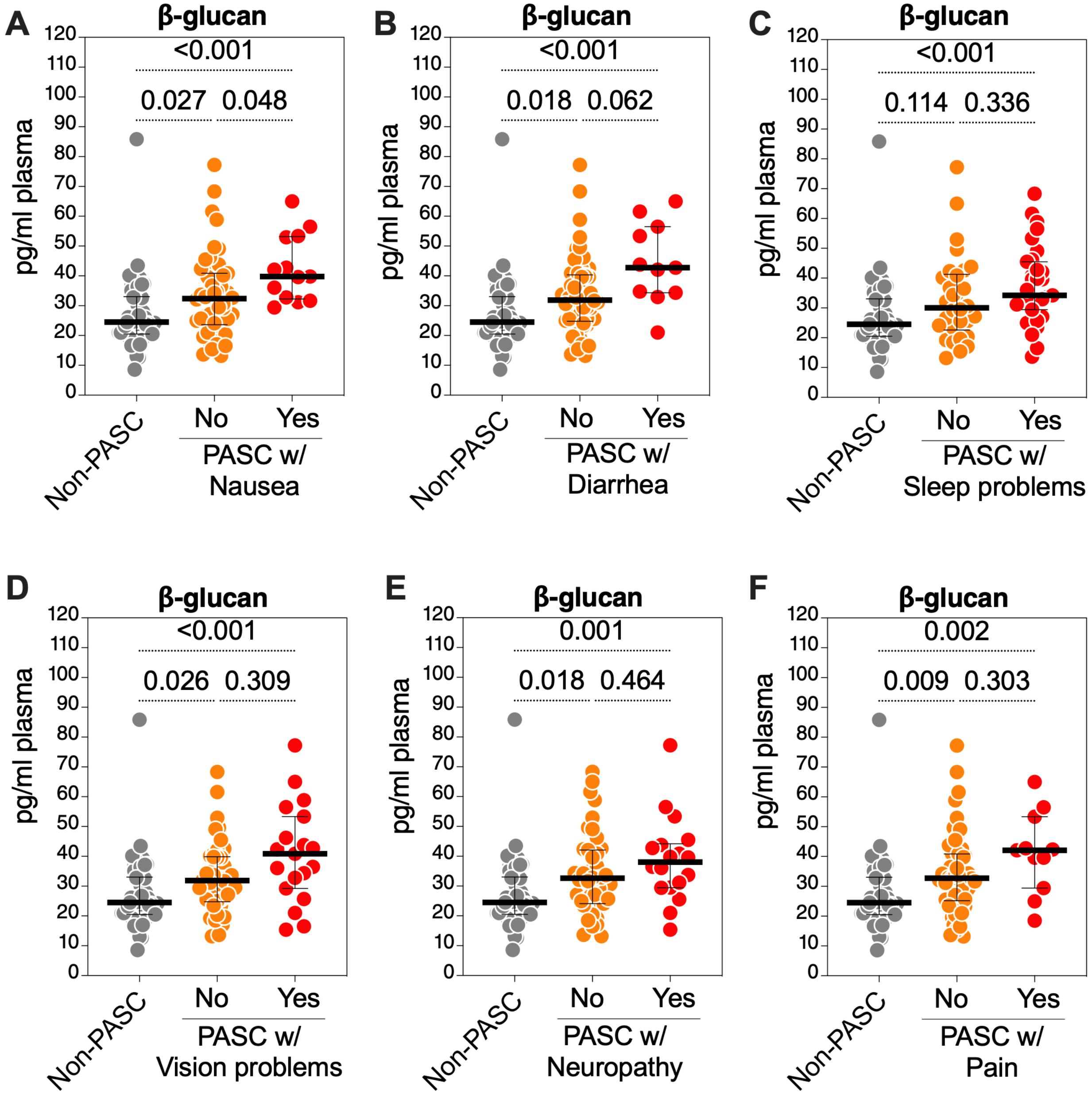

Supplementary Figure 1. β-glucan levels in plasma correlate with certain PASC symptoms. Kruskal-Wallis tests comparing β-glucan levels in non-PASC and PASC groups (from the UCSF LIINC cohort) with or without **(A)** nausea, **(B)** diarrhea, **(C)** vision problems, **(D)** sleep problems, **(E)** neuropathy, or **(F)** pain. Median and IQR are displayed.

Supplementary Figure 2

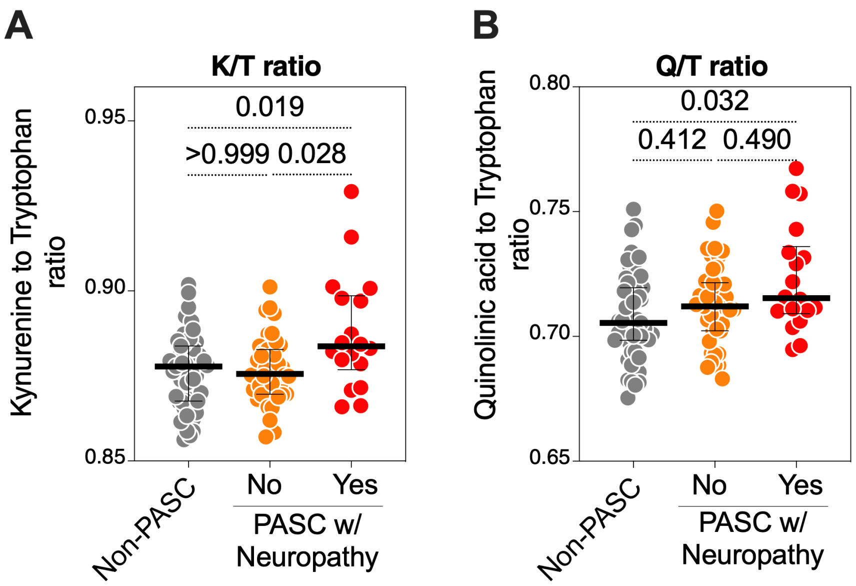

Supplementary Figure 2. K/T and Q/T ratios correlate with neuropathy during PASC. Kruskal-Wallis tests comparing **(A)** K/T ratio and **(B)** Q/T ratio between non-PASC and PASC groups (from the UCSF LIINC cohort) with or without neuropathy symptoms. Median and IQR are displayed.

Supplementary Figure 3

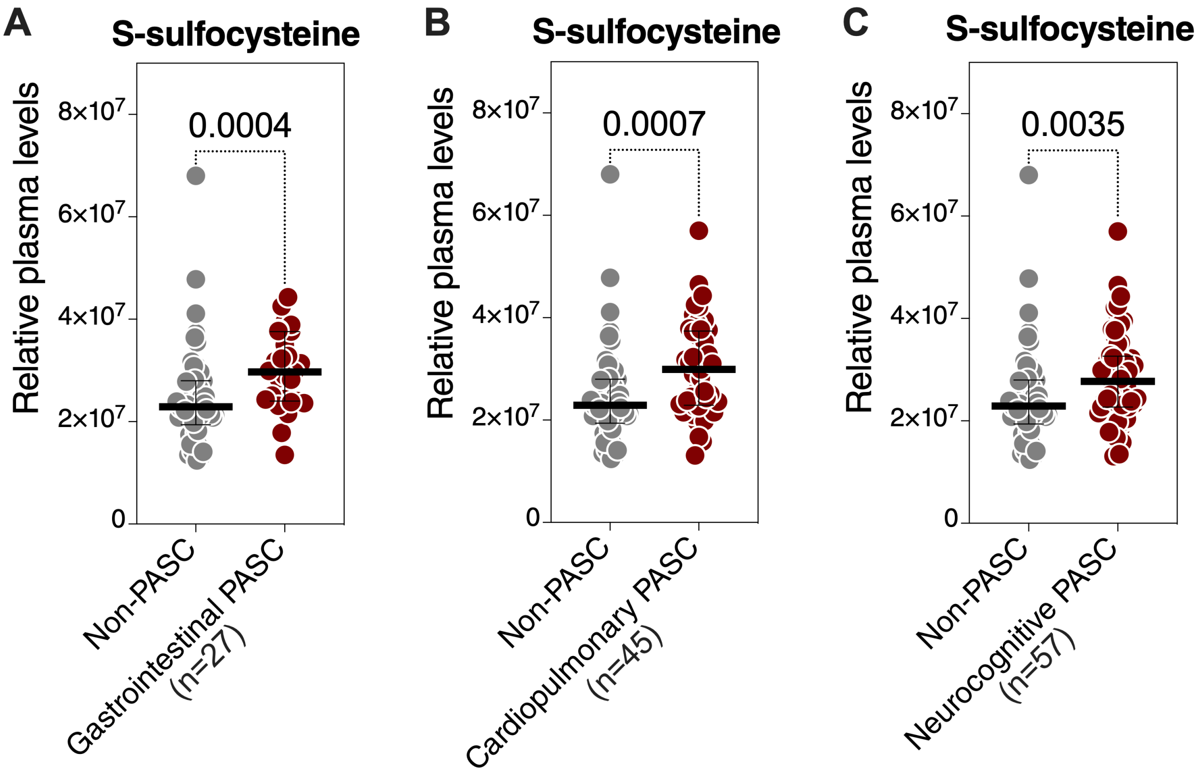

Supplementary Figure 3. Levels of plasma S-sulfocysteine are higher in individuals experiencing three PASC phenotypes, based on clinical symptom clusters, compared to those without PASC. PASC was divided to three phenotypes based on clinical symptom clusters defined as having at least one symptom in the cluster – gastrointestinal (nausea, diarrhea, loss of appetite, abdominal pain, vomiting), cardiopulmonary (cough, dyspnea, chest pain, palpitations), and neurocognitive (headache, concentration problems, dizziness, balance problems, neuropathy, vision problems). S-sulfocysteine levels were higher in individuals experiencing each of the three PASC symptom clusters compared to individuals who are not experiencing PASC. Mann–Whitney U tests. Median and IQR are displayed.

Supplementary Figure 4

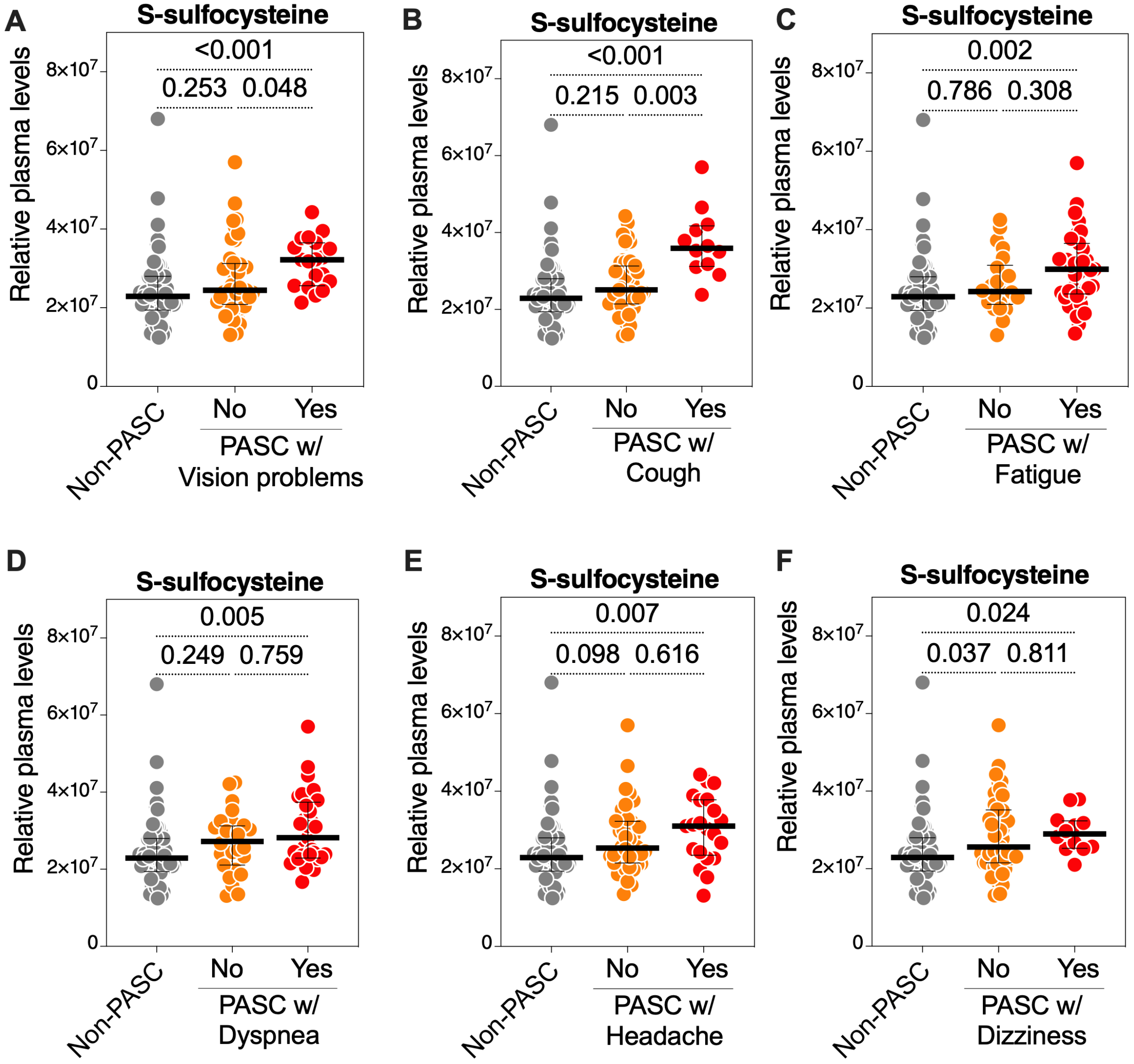

Supplementary Figure 4. S-sulfocysteine levels in plasma correlate with certain PASC symptoms. Kruskal-Wallis tests comparing S-sulfocysteine levels in non-PASC and PASC groups (from the UCSF LIINC cohort) with or without **(A)** vision problems, **(B)** fatigue, **(C)** headache, **(D)** dizziness, **(E)** cough, or **(F)** dyspnea. Median and IQR are displayed.

Supplementary Table 1. Plasma metabolites differ between PASC and non-PASC participants in the LIINC cohort.

	Marker	PASC	n	mean*	SD	SEM	min	25%	median	75%	max	Wilcoxon rank-sum *P* value
Higher in PASC than no-PASC	**S-sulfocysteine**	No	55	24.48	0.47	0.06	23.56	24.21	24.45	24.74	26.02	0.0025
		Yes	61	24.71	0.44	0.06	23.64	24.44	24.72	24.97	25.76	
	**L-Cystine**	No	55	27.35	0.52	0.07	25.93	26.99	27.35	27.69	28.58	0.0046
		Yes	61	27.63	0.51	0.06	26.34	27.31	27.66	28.01	28.49	
	**L-Glutamine**	No	55	32.88	0.19	0.03	32.35	32.77	32.87	33.01	33.36	0.0333
		Yes	61	32.97	0.24	0.03	32.45	32.85	32.95	33.15	33.49	
	**Quinolinic acid**	No	55	21.78	0.52	0.07	20.73	21.42	21.71	22.14	23.41	0.0352
		Yes	61	21.98	0.50	0.06	21.01	21.61	21.90	22.28	23.39	
	**Ornithine**	No	55	29.78	0.26	0.03	29.32	29.56	29.81	29.99	30.37	0.0387
		Yes	61	29.89	0.27	0.03	29.26	29.73	29.86	30.08	30.38	
	**2-Phenylphenol**	No	55	20.29	1.56	0.21	18.66	19.43	20.27	20.65	30.47	0.0392
		Yes	61	20.76	2.27	0.29	18.49	19.74	20.64	20.83	30.96	
Lower in PASC than no-PASC	**Sarcosine**	No	55	26.48	0.27	0.04	25.67	26.30	26.47	26.67	27.09	0.0058
		Yes	61	26.36	0.26	0.03	25.71	26.18	26.34	26.54	27.26	
	**1-Methylnicotinamide**	No	55	24.02	1.08	0.15	21.35	23.40	23.91	24.72	27.46	0.0174
		Yes	61	23.66	0.94	0.12	22.04	23.13	23.59	24.10	27.95	
	**3,5-Dihydroxybenzoic acid**	No	55	23.19	1.24	0.17	19.93	22.25	23.16	24.19	25.88	0.0376
		Yes	61	22.72	1.23	0.16	20.09	21.66	22.79	23.45	25.59	
	**Indole-3-lactic acid**	No	55	25.32	0.48	0.06	24.25	25.03	25.28	25.55	26.89	0.0436
		Yes	61	25.11	0.51	0.07	23.07	24.75	25.16	25.40	26.10	
	**L-Methionine**	No	55	30.18	0.31	0.04	28.94	29.98	30.22	30.38	30.76	0.0408
		Yes	61	30.07	0.33	0.04	29.06	29.90	30.09	30.26	30.93	
	**Pipecolic acid**	No	55	27.66	0.99	0.13	26.17	26.98	27.45	28.27	30.91	0.0430
		Yes	61	27.38	1.16	0.15	25.43	26.64	27.07	27.93	31.43	

* Log_2 transformed data

SD = Standard deviation

SEM= Standard Error of the Mean

Supplementary Table 2. Correlations between metabolites and clinical and inflammatory markers in the LIINC cohort.

			Spearman correlations among all patients										
	Markers	Parameter	# of symptoms	QoL score	GFAP	NFL	MCP1	IFNα	IFNγ	IL-6	IL-10	TNFα	IP-10
Higher in PASC than no-PASC	S-sulfocysteine	*rho*	0.3474	-0.0975	-0.1653	-0.1215	0.0594	-0.1517	-0.0221	0.1287	-0.1154	0.1906	0.0980
		P value	0.0001	0.3925	0.1377	0.2770	0.5960	0.6048	0.8495	0.2493	0.3020	0.0863	0.3842
		n	116	79	82	82	82	14	76	82	82	82	81
	L-Cystine	*rho*	0.3145	-0.2378	-0.0607	0.0521	0.1829	-0.3451	-0.0887	0.5489	0.1334	0.4746	0.1469
		P value	0.0006	0.0348	0.5882	0.6418	0.1001	0.2269	0.4462	<.0001	0.2320	<.0001	0.1907
		n	116	79	82	82	82	14	76	82	82	82	81
	L-Glutamine	*rho*	0.1950	0.0173	0.1811	-0.0923	-0.1799	0.1912	0.0394	0.0245	0.1196	-0.0188	-0.0792
		P value	0.0360	0.8795	0.1035	0.4094	0.1057	0.5126	0.7352	0.8269	0.2845	0.8667	0.4823
		n	116	79	82	82	82	14	76	82	82	82	81
	Quinolinic acid	*rho*	0.2091	-0.2667	-0.1205	0.1882	0.2445	-0.2615	0.0439	0.4423	0.1934	0.5146	0.2732
		P value	0.0243	0.0175	0.2809	0.0904	0.0268	0.3664	0.7067	<.0001	0.0817	<.0001	0.0136
		n	116	79	82	82	82	14	76	82	82	82	81
	Ornithine	*rho*	0.1815	0.0064	-0.1242	0.0941	0.1028	0.0593	0.0809	-0.0201	-0.2537	0.0267	0.0309
		P value	0.0512	0.9557	0.2661	0.4007	0.3582	0.8403	0.4871	0.8579	0.0215	0.8120	0.7839
		n	116	79	82	82	82	14	76	82	82	82	81
	2-Phenylphenol	*rho*	0.1608	0.0604	-0.0130	-0.0589	-0.0221	-0.0506	-0.0198	0.0013	-0.0080	0.0000	-0.0226
		P value	0.0847	0.5969	0.9077	0.5991	0.8436	0.8637	0.8654	0.9909	0.9433	0.9998	0.8412
		n	116	79	82	82	82	14	76	82	82	82	81
Lower in PASC than no-PASC	Sarcosine	*rho*	-0.2371	0.1424	0.2452	-0.0982	0.0741	0.0637	0.0435	0.1671	0.2102	0.1017	0.0972
		P value	0.0104	0.2105	0.0264	0.3802	0.5083	0.8286	0.7094	0.1336	0.0581	0.3631	0.3881
		n	116	79	82	82	82	14	76	82	82	82	81
	1-Methylnicotinamide	*rho*	-0.1806	0.1084	0.0638	-0.0247	-0.0501	0.0725	0.0543	-0.2808	0.0299	-0.2962	-0.0526
		P value	0.0524	0.3415	0.5690	0.8255	0.6546	0.8054	0.6414	0.0106	0.7899	0.0069	0.6408
		n	116	79	82	82	82	14	76	82	82	82	81
	3,5-Dihydroxybenzoic acid	*rho*	-0.2099	0.1481	0.0416	0.2838	0.2055	0.1121	-0.0101	-0.0774	-0.0548	-0.0526	-0.0222
		P value	0.0237	0.1927	0.7108	0.0098	0.0640	0.7028	0.9311	0.4895	0.6249	0.6390	0.8439
		n	116	79	82	82	82	14	76	82	82	82	81
	Indole-3-lactic acid	*rho*	-0.2076	0.0977	0.0622	0.0232	-0.0166	0.4066	-0.0724	-0.2459	-0.0029	-0.2940	-0.1782
		P value	0.0253	0.3915	0.5789	0.8360	0.8822	0.1491	0.5343	0.0259	0.9795	0.0073	0.1115
		n	116	79	82	82	82	14	76	82	82	82	81
	L-Methionine	*rho*	-0.1406	0.1086	0.1052	-0.0750	0.0688	-0.3451	0.0985	0.1044	0.1520	0.0682	0.1501
		P value	0.1322	0.3410	0.3467	0.5028	0.5391	0.2269	0.3973	0.3508	0.1727	0.5426	0.1811
		n	116	79	82	82	82	14	76	82	82	82	81
	Pipecolic acid	*rho*	-0.1812	0.0697	0.0161	-0.2210	-0.0622	0.2352	-0.0250	-0.2557	0.0239	-0.2046	0.0811
		P value	0.0516	0.5418	0.8860	0.0460	0.5788	0.4183	0.8300	0.0204	0.8316	0.0652	0.4719
		n	116	79	82	82	82	14	76	82	82	82	81

			Spearman correlations among no-PASC patients										
	Markers	Parameter	# of symptoms	QoL score	GFAP	NFL	MCP1	IFNα	IFNγ	IL-6	IL-10	TNFα	IP-10
Higher in PASC than no-PASC	S-sulfocysteine	*rho*		-0.1051	-0.1909	0.0320	0.3566		0.1019	0.1053	0.0178	0.0716	0.1245
		P value		0.5543	0.2579	0.8508	0.0303		0.5724	0.5352	0.9168	0.6737	0.4630
		n		34	37	37	37		33	37	37	37	37
	L-Cystine	*rho*		-0.0484	-0.0756	0.2354	0.2909		-0.0572	0.7110	-0.0121	0.4772	0.2776
		P value		0.7859	0.6564	0.1607	0.0807		0.7521	<.0001	0.9434	0.0028	0.0962
		n		34	37	37	37		33	37	37	37	37
	L-Glutamine	*rho*		-0.0069	0.1560	0.0624	-0.1313		-0.2386	-0.0218	0.2385	-0.0007	-0.2224
		P value		0.9691	0.3565	0.7139	0.4384		0.1811	0.8980	0.1552	0.9967	0.1859
		n		34	37	37	37		33	37	37	37	37
	Quinolinic acid	*rho*		0.0847	-0.0299	0.3092	0.3075		0.0488	0.4829	0.0413	0.4054	0.3376
		P value		0.6341	0.8607	0.0626	0.0641		0.7874	0.0025	0.8085	0.0128	0.0410
		n		34	37	37	37		33	37	37	37	37
	Ornithine	*rho*		-0.0961	-0.0704	-0.2184	0.2053		0.2253	0.0773	-0.1859	0.1577	0.1055
		P value		0.5887	0.6788	0.1942	0.2228		0.2075	0.6493	0.2707	0.3514	0.5343
		n		34	37	37	37		33	37	37	37	37
	2-Phenylphenol	*rho*		0.1387	0.1719	-0.0505	0.0688		-0.1043	-0.0213	0.1098	-0.2079	0.0775
		P value		0.4341	0.3090	0.7666	0.6860		0.5636	0.9002	0.5178	0.2169	0.6483
		n		34	37	37	37		33	37	37	37	37
Lower in PASC than no-PASC	Sarcosine	*rho*		-0.1135	0.1989	0.0673	0.1726		0.0521	0.1916	0.2504	0.0763	0.0481
		P value		0.5226	0.2379	0.6921	0.3070		0.7732	0.2560	0.1350	0.6534	0.7773
		n		34	37	37	37		33	37	37	37	37
	1-Methylnicotinamide	*rho*		0.0069	-0.1131	-0.0695	-0.0211		0.0371	-0.4007	-0.0837	-0.3400	-0.1380
		P value		0.9691	0.5051	0.6829	0.9014		0.8376	0.0140	0.6224	0.0395	0.4154
		n		34	37	37	37		33	37	37	37	37
	3,5-Dihydroxybenzoic acid	*rho*		0.0669	0.1155	0.2814	0.1679		-0.0849	-0.0059	-0.0197	0.0766	0.1375
		P value		0.7070	0.4962	0.0915	0.3207		0.6386	0.9722	0.9080	0.6524	0.4170
		n		34	37	37	37		33	37	37	37	37
	Indole-3-lactic acid	*rho*		0.3240	0.0974	-0.2909	-0.0194		-0.1544	-0.2764	0.0026	-0.4524	-0.2755
		P value		0.0616	0.5662	0.0807	0.9091		0.3909	0.0977	0.9878	0.0049	0.0989
		n		34	37	37	37		33	37	37	37	37
	L-Methionine	*rho*		-0.0302	-0.0832	0.2141	0.2350		0.0050	0.3755	0.1183	0.3523	0.4028
		P value		0.8656	0.6244	0.2033	0.1616		0.9779	0.0220	0.4856	0.0325	0.0134
		n		34	37	37	37		33	37	37	37	37
	Pipecolic acid	*rho*		0.0501	-0.1057	-0.1114	-0.1724		-0.0147	-0.2598	-0.0311	-0.2482	0.0804
		P value		0.7784	0.5334	0.5115	0.3077		0.9353	0.1204	0.8552	0.1385	0.6363
		n		34	37	37	37		33	37	37	37	37

			Spearman correlations among PASC patients										
	Markers	Parameter	# of symptoms	QoL score	GFAP	NFL	MCP1	IFNα	IFNγ	IL-6	IL-10	TNFα	IP-10
Higher in PASC than no-PASC	S-sulfocysteine	*rho*	0.3072	0.1592	-0.0975	-0.1992	0.0021	-0.3727	-0.0862	0.0607	-0.1841	0.1556	0.0677
		P value	0.0160	0.2963	0.5240	0.1895	0.9890	0.2589	0.5824	0.6919	0.2262	0.3074	0.6626
		n	61	45	45	45	45	11	43	45	45	45	44
	L-Cystine	*rho*	0.2431	-0.0994	-0.0302	-0.0501	0.1943	-0.4636	-0.0807	0.4224	0.2148	0.4605	-0.0065
		P value	0.0590	0.5161	0.8440	0.7440	0.2008	0.1509	0.6069	0.0038	0.1566	0.0015	0.9667
		n	61	45	45	45	45	11	43	45	45	45	44
	L-Glutamine	*rho*	0.0521	0.1797	0.2551	-0.1751	-0.2115	-0.0273	0.1701	-0.0229	0.0285	-0.1073	-0.0795
		P value	0.6902	0.2375	0.0908	0.2500	0.1632	0.9366	0.2756	0.8812	0.8528	0.4832	0.6080
		n	61	45	45	45	45	11	43	45	45	45	44
	Quinolinic acid	*rho*	0.0951	-0.2555	-0.0862	0.1748	0.2849	-0.5818	0.0745	0.3391	0.3229	0.5725	0.1821
		P value	0.4662	0.0902	0.5736	0.2507	0.0579	0.0604	0.6351	0.0227	0.0305	<.0001	0.2368
		n	61	45	45	45	45	11	43	45	45	45	44
	Ornithine	*rho*	0.0065	0.1758	-0.1294	0.3322	0.0603	-0.0182	0.0360	-0.1322	-0.3183	-0.1170	-0.0665
		P value	0.9604	0.2481	0.3970	0.0258	0.6938	0.9577	0.8186	0.3869	0.0331	0.4440	0.6679
		n	61	45	45	45	45	11	43	45	45	45	44
	2-Phenylphenol	*rho*	-0.0436	0.0994	-0.0730	-0.0999	-0.0623	0.0818	0.0527	-0.0600	-0.0974	0.0307	-0.1915
		P value	0.7387	0.5158	0.6337	0.5139	0.6842	0.8110	0.7371	0.6957	0.5246	0.8413	0.2129
		n	61	45	45	45	45	11	43	45	45	45	44
Lower in PASC than no-PASC	Sarcosine	*rho*	0.0073	-0.0119	0.2278	-0.2235	-0.0038	0.1546	0.1009	0.2335	0.1867	0.1942	0.1550
		P value	0.9556	0.9383	0.1323	0.1401	0.9801	0.6500	0.5198	0.1227	0.2195	0.2011	0.3149
		n	61	45	45	45	45	11	43	45	45	45	44
	1-Methylnicotinamide	*rho*	0.0620	-0.0389	0.0490	-0.0119	-0.1702	0.0091	0.0739	-0.1530	0.0735	-0.2111	0.0660
		P value	0.6350	0.7998	0.7492	0.9384	0.2636	0.9788	0.6379	0.3158	0.6313	0.1640	0.6705
		n	61	45	45	45	45	11	43	45	45	45	44
	3,5-Dihydroxybenzoic acid	*rho*	-0.1318	-0.0185	-0.0386	0.2648	0.1444	-0.1273	0.0337	-0.1228	-0.0842	-0.0949	-0.1834
		P value	0.3113	0.9038	0.8012	0.0787	0.3440	0.7092	0.8302	0.4216	0.5824	0.5354	0.2335
		n	61	45	45	45	45	11	43	45	45	45	44
	Indole-3-lactic acid	*rho*	-0.1110	-0.1940	0.0003	0.2206	-0.0258	0.4364	-0.0253	-0.2294	0.0258	-0.1622	-0.1015
		P value	0.3943	0.2017	0.9986	0.1454	0.8663	0.1797	0.8721	0.1296	0.8663	0.2871	0.5122
		n	61	45	45	45	45	11	43	45	45	45	44
	L-Methionine	*rho*	0.1284	0.0320	0.2609	-0.3156	-0.1560	-0.2546	0.1821	0.0460	0.1947	-0.0531	0.0312
		P value	0.3243	0.8347	0.0835	0.0347	0.3062	0.4500	0.2424	0.7642	0.1999	0.7290	0.8409
		n	61	45	45	45	45	11	43	45	45	45	44
	Pipecolic acid	*rho*	-0.0095	-0.1221	0.0755	-0.2920	-0.1183	0.4091	-0.0486	-0.2242	0.0066	-0.1571	0.0599
		P value	0.9423	0.4243	0.6221	0.0516	0.4389	0.2115	0.7572	0.1386	0.9657	0.3029	0.6993
		n	61	45	45	45	45	11	43	45	45	45	44

Red = significant positive correlation
Blue = significant negative correlation
